# Glycolysis Inhibition Induces Functional and Metabolic Exhaustion of CD4^+^ T Cells in Type 1 Diabetes

**DOI:** 10.3389/fimmu.2021.669456

**Published:** 2021-06-07

**Authors:** Christina P. Martins, Lee A. New, Erin C. O’Connor, Dana M. Previte, Kasey R. Cargill, Isabelle L. Tse, Sunder Sims- Lucas, Jon D. Piganelli

**Affiliations:** ^1^ Department of Infectious Diseases and Microbiology, Graduate School of Public Health, University of Pittsburgh, Pittsburgh, PA, United States; ^2^ Department of Pediatric Surgery, Rangos Research Center, UPMC Children’s Hospital of Pittsburgh, University of Pittsburgh, Pittsburgh, PA, United States; ^3^ Department of Pediatrics, Rangos Research Center, UPMC Children’s Hospital of Pittsburgh, University of Pittsburgh, Pittsburgh, PA, United States

**Keywords:** glycolysis, type 1 diabetes, immunometabolism, T cell exhaustion, autoimmunity, PD-1, LAG-3

## Abstract

In Type 1 Diabetes (T1D), CD4^+^ T cells initiate autoimmune attack of pancreatic islet β cells. Importantly, bioenergetic programs dictate T cell function, with specific pathways required for progression through the T cell lifecycle. During activation, CD4^+^ T cells undergo metabolic reprogramming to the less efficient aerobic glycolysis, similarly to highly proliferative cancer cells. In an effort to limit tumor growth in cancer, use of glycolytic inhibitors have been successfully employed in preclinical and clinical studies. This strategy has also been utilized to suppress T cell responses in autoimmune diseases like Systemic Lupus Erythematosus (SLE), Multiple Sclerosis (MS), and Rheumatoid Arthritis (RA). However, modulating T cell metabolism in the context of T1D has remained an understudied therapeutic opportunity. In this study, we utilized the small molecule PFK15, a competitive inhibitor of the rate limiting glycolysis enzyme 6-phosphofructo-2-kinase/fructose-2,6- biphosphatase 3 (PFKFB3). Our results confirmed PFK15 inhibited glycolysis utilization by diabetogenic CD4^+^ T cells and reduced T cell responses to β cell antigen *in vitro*. In an adoptive transfer model of T1D, PFK15 treatment delayed diabetes onset, with 57% of animals remaining euglycemic at the end of the study period. Protection was due to induction of a hyporesponsive T cell phenotype, characterized by increased and sustained expression of the checkpoint molecules PD-1 and LAG-3 and downstream functional and metabolic exhaustion. Glycolysis inhibition terminally exhausted diabetogenic CD4^+^ T cells, which was irreversible through restimulation or checkpoint blockade *in vitro* and *in vivo*. In sum, our results demonstrate a novel therapeutic strategy to control aberrant T cell responses by exploiting the metabolic reprogramming of these cells during T1D. Moreover, the data presented here highlight a key role for nutrient availability in fueling T cell function and has implications in our understanding of T cell biology in chronic infection, cancer, and autoimmunity.

## Introduction

Invasion of pancreatic islets by immune cells is a hallmark of Type 1 Diabetes (T1D), where the innate and adaptive immune systems work cooperatively to mediate damage of insulin- secreting β cells (1, 2). This attack is largely orchestrated by self-reactive CD4^+^ T cells, which are fundamental drivers of disease pathology (2). During their life cycle, CD4^+^ T cells rely on specific metabolic pathways to generate energy in the form of adenosine triphosphate (ATP) (3, 4). However, it has become abundantly clear that these programs are not merely for energy production, but rather are necessary for the ability of T cells to carry out specialized effector capabilities, including interferon gamma (IFNγ) secretion (5). During homeostasis, naïve CD4^+^ T cells utilize oxidative phosphorylation (OXPHOS) to support surveillance efforts and migration in the periphery. Upon encounter with antigen (i.e., islet β cells in T1D), activated T cells undergo robust metabolic reprogramming marked by a transition to the less efficient aerobic glycolysis (3, 4). Although net energy obtained through the glycolysis pathway is far less than what is generated *via* OXPHOS (net gain of 2 versus approximately 36 ATP molecules, respectively), glycolysis is required to generate ATP quickly to support T cell activation, clonal expansion, and effector cytokine production (5).

While distinct metabolic programs dictate T cell differentiation and function, a number of studies have also implicated nutrient availability as an important determinant of T cell fitness. This is especially evident in the tumor microenvironment (TME), where reports have demonstrated that cancer cells outcompete T cells for key nutrients like glucose and amino acids, which are required for acquisition of specialized effector functions (6). This battle for metabolic substrates, along with persistent antigen exposure, has been implicated in suppressing immune responses and driving T cell exhaustion. Exhaustion is defined as a state of dysfunction characterized by increased expression of immune inhibitory receptors (IRs) like programmed cell death protein- 1 (PD-1) and Lymphocyte Activation Gene-3 (LAG-3), and a marked decrease in T cell effector functions that allow tumors to go unabated by the immune system (6, 7). Although metabolic insufficiencies demonstrate a major hurdle in reinvigorating tumor-specific T cell responses, the opposite is plausible in settings of autoimmunity, where enforcing T cell exhaustion by targeting metabolism may be a novel mechanism by which tolerance to self-antigens is restored (8).

Efforts to target the glycolysis pathway have been successfully employed to limit tumor growth and metastasis in cancer, with a number of these inhibitors undergoing FDA clinical trials (9–12). One such inhibitor, PFK15, is a small molecule inhibitor of 6-phosphofructo-2-kinase/fructose-2,6- biphosphatase 3 (PFKFB3), an enzyme involved in a cells commitment to metabolize glucose *via* glycolysis (9, 12, 13). Regarding autoimmunity, researchers have successfully targeted metabolism as a means to control T cell responses in models of Systemic Lupus Erythematosus (SLE), Multiple Sclerosis (MS), and Rheumatoid Arthritis (RA), however the ability to target metabolic pathways to control T1D have been largely unstudied (14–17). Previously, our laboratory demonstrated that redox modulation *via* disruption of third signal reactive oxygen species (ROS) impeded T cell metabolic reprogramming to glycolysis, and altered the diabetogenic potential of autoreactive CD4^+^ T cells (18). However, whether specifically modulating the glycolysis pathway could be used to limit the activation of autoreactive CD4^+^ T cells and prevent attack of pancreatic β cells deserves further exploration. Based on these previous studies, we hypothesized that use of the glycolysis inhibitor PFK15 would inhibit the activation, proliferation, and effector capabilities of autoreactive CD4^+^ T cells, thereby delaying the onset of T1D. Herein, we demonstrate that PFK15 treatment interrupts metabolic reprogramming to glycolysis upon activation with β cell antigen, and reduces T cell responses *in vitro*, while delaying the onset of T1D *in vivo*. These results were, in part, due to increased and sustained expression of checkpoint molecules PD-1 and LAG-3 and downstream functional and metabolic exhaustion of diabetogenic T cell clones. These findings support that inhibition of glycolysis drives T cell exhaustion, and that metabolic modulation may serve as a novel therapeutic target to control T cell metabolism and restore tolerance in autoimmunity.

## Materials and Methods

### Animals

Non-obese diabetic (NOD), NOD.BDC2.5.TCR.Tg (BDC2.5) and NOD.*scid* mice were maintained under specific pathogen free conditions in the animal facility located at the Rangos Research Center within UPMC Children’s Hospital of Pittsburgh. All animal experiments were approved by the University of Pittsburgh’s Institutional Animal Care and Use Committee (IACUC; Assurance Number: D16-00118). Male and female mice aged 6-12 weeks old were used in all experiments.

### Splenocyte Isolation and *In Vitro* Stimulation

BDC2.5 animals were sacrificed, and spleens harvested and homogenized into single cell suspensions as described (18). Red blood cells were lysed using RBC lysis buffer (Sigma Aldrich). For *in vitro* experiments, 2.5 x 10^6^ splenocytes were plated per well in 24 well plates and stimulated with 0.05 µM of their peptide mimotope ± 5 µM PFK15 soluble drug (Selleck), 0.2-1 mM 2-DG (Sigma Aldrich), 25-50 µM YN1 (Millipore Sigma), or 2.5-5 µM PFK158 (Selleck) for 24-72 hours. Cells and culture supernatants were collected for downstream flow cytometry, western blotting, ELISA, and lactate measurement analyses.

### Flow Cytometry

5x10^5^ – 1x10^6^ cells were harvested at indicated timepoints and surface stained for flow cytometric analysis as described (18). Briefly, cells were incubated with Fc block (CD16/CD32; BD Biosciences) for 15 minutes prior to staining for flow cytometry. Surface staining was performed at 4°C using CD4-PE, PerCP-Cy5.5, or APC (Clone RM4-5), CD69-PeCy7 (Clone H1.2F3), CD25-APC (Clone PC61), CD223-PE (LAG-3; Clone C9B7W), CD279-BV480 (PD-1; Clone J43) antibodies (BD Biosciences) in FACS buffer (1% BSA in PBS). For proliferation measurements, splenocytes were labeled with Cell Proliferation Dye Violet (BD Bioscience) per manufacturer’s instructions prior to stimulation. After indicated timepoints, cells were harvested, and surface stained as described above. In some instances, cells were fixed in 2% PFA for 15 minutes at 4°C (Thermo Fisher Scientific). Cells were stored at 4°C until time of analysis.

To measure glucose uptake, splenocytes were incubated with 100 μM of the fluorescent glucose analog 2-(*N*-(7-Nitrobenz-2-oxa-1,3-diazol-4-yl) Amino)-2-Deoxyglucose (2-NBDG; Cayman Chemical) for 10 minutes at 37°C prior to harvest as described (18, 19). Cells were washed with PBS and surface stained for CD4 expression and analyzed live by flow cytometry. To measure fatty acid uptake, cells were harvested, surface stained for CD4 expression, and incubated with the fluorescent fatty acid BODIPY FL C16 (4,4-Difluoro-5,7-Dimethyl-4-Bora-3a,4a-Diaza-*s*-Indacene-3-Hexadecanoic Acid; Invitrogen) in serum-free warm PBS for 30 minutes at 37°C. Cells were washed with PBS and analyzed live by flow cytometry.

To assess mitochondrial function, Day 14 BDC2.5 T cell clones were harvested, surface stained for CD4 expression, and incubated with MitoTracker green, MitoSOX red, or TMRE (tetramethylrhodamine, ethyl ester, perchlorate; Invitrogen) in warm PBS at 37°C for 15-30 minutes. Cells were washed with PBS and analyzed live by flow cytometry as described previously (19). For all flow cytometry studies, fluorescence was measured using a FACS Aria II flow cytometer (BD Biosciences). All data were analyzed using FlowJo software (v10.5.3) and samples were gated on CD4^+^ cells. Forward scatter of CD4^+^ T cells was also determined by flow cytometry.

### Preparation of Protein Lysates and Western Blotting

Cells were lysed by sonication in anti-pY lysis buffer (50 mM Tris pH 8.0, 137 mM NaCl, 10% glycerol, 1% NP-40, 1 mM NaF, 10 μg/ml leupeptin, 10 μg/ml aprotinin, 2 mM Na_3_VO_4_, and 1 mM PMSF). Protein concentration was determined by Bicinchoninic acid protein (BCA) assay (Thermo Fisher Scientific). 25 μg of protein per sample were boiled in 6x Lammaeli buffer (BIORAD) for 5 minutes and separated on 4-20% gradient SDS-PAGE gels (BIORAD). Samples were then transferred to PVDF membranes for 2 hours in 3% MeOH Tris-Glycine Transfer buffer (BIORAD). Western blots were blocked in 5% non-fat dry milk in Tris-buffered Saline with 1% Tween-20 (TBST). Blots were probed with the following antibodies in 5% BSA/TBST overnight at 4°C: Glut-1, CPT1α (1:1000; Abcam), HK2, PFKFB3, LDHA (1:1000; Cell Signaling), and β-actin as a loading control (1: 10,000; Sigma- Aldrich). Membranes were washed with TBST and incubated with HRP-conjugated goat anti-rabbit or rabbit anti-mouse (Jackson ImmunoResearch 1:10,000) secondary antibodies for 2 hrs in 5% non- fat milk/TBST at room temperature. Chemiluminescence was detected using SuperSignal West Pico PLUS Chemiluminescence solution (Thermo Fisher Scientific) and the iBright FL1500 imaging system (Invitrogen).

### Lactate and Cytokine Measurements

Cell culture supernatants from *in vitro* experiments were harvested and used to measure IL-2, IFNγ, and TNFα by ELISA. Antibody pairs for IFNγ and IL-2 ELISAs were purchased from BD Biosciences, and TNFα ELISA kits were purchased from R&D according to the manufacturer’s instructions. ELISAs were read on a SpectraMax M2 microplate reader (Molecular Devices) and data analyzed using SoftMax Pro version 7.0.2 software (Molecular Devices). Lactate, a byproduct of aerobic glycolysis, was measured using the Lactate Plus meter and test strips per manufacturer’s instructions (Novus Biologics).

### CD4^+^ T Cell Isolation and *Ex Vivo* Activation and Expansion

Spleens from BDC2.5 animals were harvested and homogenized into single cell suspensions as described above. CD4^+^ T cells were isolated by magnetic bead separation using the EasySep CD4^+^ T cell Negative Selection isolation kit (StemCell) per the manufacturer’s instructions. For *ex vivo* activation, 6 well plates were coated with plate bound αCD3 (BD Biosciences; 1 ug/mL) in PBS for at least 3 hours in a cell culture incubator (37° C, 5% CO_2_). The antibody solution was decanted, and 5x10^6^ isolated cells were plated with 100 U/mL IL-2 and 1 ug/mL soluble αCD28 (BD Biosciences) for 3 days in a cell culture incubator. After 3 days in culture, cells were harvested and transferred to T-75 flasks with an additional 100 U/mL IL-2 for expansion. At the end of the 3-day expansion in IL-2, cells were isolated, counted, and washed with sterile PBS for adoptive transfer experiments.

### Adoptive Transfer Model of T1D

1 x 10^7^ *ex vivo* activated CD4^+^ T cells from BDC2.5 mice (described above) were injected i.p. into NOD.*scid* recipients. For initial prevention studies, recipient animals were split into two groups. One cohort of recipients received 25 mg/kg PFK15 (Selleck) dissolved in 5% DMSO + 45% PEG300 + 1% Tween80 + 49% ddH_2_O prepared fresh; the other cohort received vehicle control every other day for 2 weeks. In reversibility studies, recipient animals were placed into one of the following treatment groups: 1) Vehicle Control, 2) PFK15 + IgG (Isotype controls for αPD-1 and αLAG-3 blocking antibodies; 200 μg each per treatment), or 3) PFK15 + 200 μg αPD-1 (clone J43; BioXCell), + 200 μg αLAG-3 (clone C9B7W; BioXCell) as previously described (19, 20). Animals were treated every other day for two weeks, with checkpoint blockade or IgG treatment initiated during the second week. Body weights and blood glucose (BG) levels were monitored over the course of the experiments. Animals were deemed diabetic after two consecutive BG readings ≥ 350 mg/dL. Diabetic animals were sacrificed at indicated timepoints and peripheral blood, pancreata, and spleens were harvested for downstream analyses.

### Tissue Collection and Histological Assessment

Pancreatic tissue was collected and fixed in 4% paraformaldehyde (PFA; Thermo Fisher Scientific) overnight at 4°C. Fixed tissue was processed and embedded in paraffin by the Histology Core Laboratory located at UPMC Children’s Hospital of Pittsburgh’s Rangos Research Center (21). Embedded tissue was sectioned at 4 μm thickness and stained with hematoxylin and eosin (H&E) for histological examination of immune infiltration in pancreatic islets. Samples were imaged using a Nikon Eclipse E800 microscope (Nikon) and associated software.

### Immunofluorescent Staining

Immunofluorescent staining was performed on paraffin embedded samples prepared as described above. Antigen retrieval was performed in either sodium citrate or Tris-EDTA buffers followed by overnight incubation with primary antibodies against insulin (1:100; Santa Cruz), CD3 (1:100; Abcam), and PD-1 (1:50; Abcam) all co-stained with DAPI (1:3000; Thermo Fisher Scientific). The following day, slides were incubated with Alexa Fluor 488 conjugated donkey anti- rabbit secondary antibody (Invitrogen). Samples were imaged using a Leica DMi8 inverted microscope (Leica) and LAS X Navigator software (Leica).

### Maintenance of BDC2.5 T Cell Clones

CD4^+^ MHC-II restricted BDC2.5 T cells, a generous gift from Dr. Kathryn Haskins (University of Colorado), were maintained in supplemented DMEM as previously described (22–25). Briefly, BDC2.5 T cells were cultured in T-25 flasks with β membrane (antigen), irradiated NOD splenocytes (antigen presenting cells: APC), and EL-4 supernatant (source of IL-2) for 2 weeks in a cell culture incubator. For mechanistic studies, a subset of flasks were treated with **5** µM PFK15 every third day over the course of the restimulation period. Day 8 and 14 T cells and culture supernatants were harvested for downstream analyses. Similarly, for reinvigoration studies, untreated and PFK15 treated T cells were put into restimulation flasks **±5**µg/mL αPD-1 (clone J43; BioXCell), αLAG-3 (clone C9B7W; BioXCell), or αPD-1 + αLAG-3. Cells were treated every third day for 2 weeks.

### ADP/ATP Ratio Measurements

Day 14 control and PFK15 treated T cell clones were harvested and assayed for the ADP/ATP ratio per the manufacturer’s instructions (Millipore Sigma).

### Statistical Analyses

All data are presented as mean values ± standard error of the mean (SEM), with n indicating the number of independent experiments or animals. Student’s t-test, One- way ANOVA, or Two-way ANOVA were used where appropriate. For survival studies, Kaplan-Meier analysis was used to measure significance of diabetes incidence. A p-value of p < 0.05 was considered significant for all statistical analyses. Histology and immunofluorescent images were generated using Photoshop. All statistics and graphs were generated using GraphPad Prism software.

## Results

### PFK15 Interrupts Metabolic Reprogramming to Glycolysis and Reduces T Cell Effector Functions During Activation

**Figure 1:**
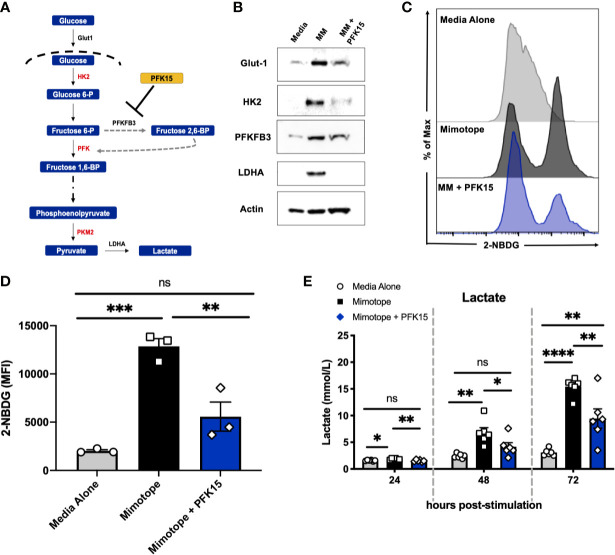
PFK15 treatment inhibits metabolic reprogramming to glycolysis during T cell activation. NOD.BDC2.5 splenocytes were stimulated with their cognate peptide MM ± PFK15 for 24-72 hrs. **(A)** Schematic diagram displaying the mechanism of action of PFK15. **(B)** Representative western blot analysis of glycolysis proteins in untreated, MM stimulated, and MM+ PFK15 treated splenocytes after 48 hrs in culture. **(C, D)** Representative histogram and statistical analysis showing 2-NBDG fluorescence and MFI of CD4^+^ T cells, indicative of glucose uptake 48 hrs post stimulation (n = 3). **(E)** Lactate measurements in cell culture supernatants (n = 6). All data are presented as the mean ± SEM. (not significant (ns), *p < 0.05, **p < 0.01, ***p < 0.005, ****p < 0.0001).

To determine the effect glycolysis inhibition would have on the activation and subsequent metabolic reprogramming of autoreactive CD4^+^ T cells in T1D, we stimulated splenocytes from NOD.BDC2.5.TCR.Tg animals *in vitro* with their cognate peptide mimotope (MM) ± PFK15 (a PFKFB3 inhibitor; **Figure 1A**), as previously described (18). PFK15 treated BDC2.5 splenocytes failed to upregulate glycolysis-associated proteins glucose transporter-1 (Glut-1), hexokinase 2 (HK2), PFKFB3, and lactate dehydrogenase A (LDHA) in response to MM stimulation compared to T cells stimulated without treatment (**Figure 1B**). Additionally, PFK15 treatment decreased glucose (2-NBDG) uptake by CD4^+^ T cells upon stimulation to levels similar to T cells in media alone (**Figures 1C, D**). A significant reduction in 2-NBDG fluorescence from treated CD4^+^ T cells was most likely due to reduced expression of Glut-1 (**Figure 1B**), indicating decreased capacity to engage in aerobic glycolysis (18, 26). Utilization of the glycolysis pathway by CD4^+^ T cells is accompanied by increased secretion of the by product lactate (3, 4, 18, 27). Splenocytes stimulated with MM alone displayed a significant increase in lactate secretion in cell culture supernatants as expected, indicating increased glycolytic flux (**Figure 1E**). In comparison, PFK15 treated splenocytes secreted less lactate compared to MM stimulated T cells, further confirming an inability to metabolically transition to glycolysis upon encounter with β cell antigen (**Figure 1E**).

**Figure 2:**
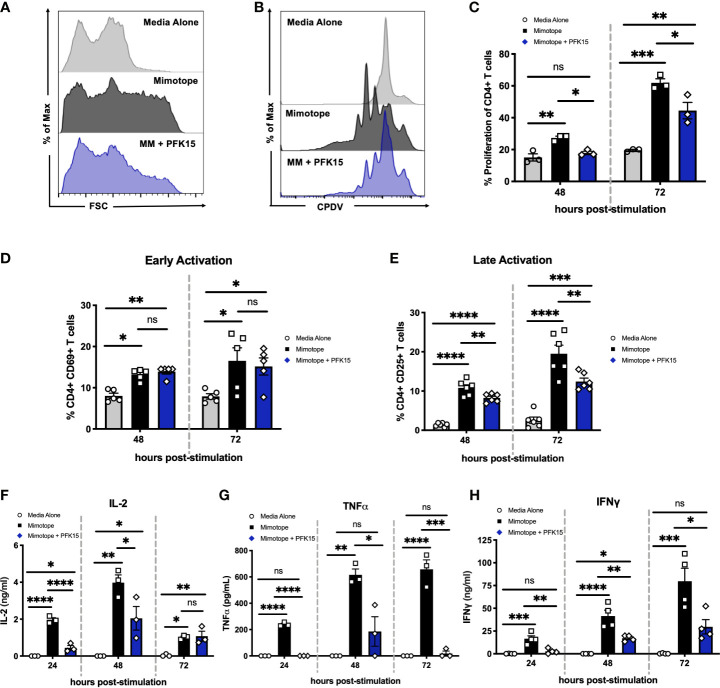
Inhibiting glycolysis suppresses CD4^+^ T cell responses to β cell antigen *in vitro*. Assessing the impact of glycolysis inhibition on diabetogenic CD4^+^ T cell responses *in vitro*. **(A)** Representative histogram measuring forward scatter (FSC). **(B, C).** Representative histogram and statistical analysis of CD4^+^ T cell proliferation assessed by cell proliferation dye violet (CPDV) dilution (n = 3). **(D)** Frequency of CD4^+^ CD69^+^ T cells (n = 5). **(E)** Frequency of CD4^+^ CD25^+^ T cells (n = 6). **(F–H)** ELISA analysis of IL-2, TNFα, and IFN*γ* in cell culture supernatants 24-72 hrs post stimulation (n = 3-4). All data are presented as the mean ± SEM. (not significant (ns), *p < 0.05, **p < 0.01, ***p < 0.005, ****p < 0.0001).

As bioenergetics and T cell function are intricately linked, we next determined the impact of glycolysis inhibition on the activation, proliferation, and effector capabilities of BDC2.5 T cells. While activated CD4^+^ T cells displayed increased forward scatter (FSC) compared to unstimulated controls, indicative of increased cell growth, PFK15 treatment resulted in reduced FSC compared to stimulated T cells, indicating inhibited growth (**Figure 2A**). We then assessed proliferative capacity by measuring cell proliferation dye violet (CPDV) dilution by CD4^+^ T cells in all three treatment groups. MM stimulated T cells displayed robust proliferation in response to antigen; however, PFK15 treatment significantly reduced this response (**Figures 2B, C**).

To interrogate the activation status of PFK15 treated T cells, expression of the early activation marker CD69, and the late activation marker and high- affinity interleukin-2 (IL-2) receptor CD25, was measured 48-72 hours post stimulation on CD4^+^ T cells by flow cytometry (**Figures 2D, E**). There were no appreciable differences in the expression of CD69 in stimulated or PFK15 treated groups indicating that PFK15 treatment does not interfere with early activation (**Figure 2D**). Conversely, CD25 expression was significantly decreased with PFK15 treatment, suggesting an inability to fully transition to late activation status when glycolysis is inhibited (**Figure 2E**). Since glycolysis is required for acquisition of effector functions (5), we interrogated this ability by kinetically measuring IL-2, tumor necrosis factor alpha (TNFα), and IFNγ in cell culture supernatants (**Figures 2F–H**). ELISA analysis revealed a reduced ability to secrete all three cytokines. The phenotype we observed was due to specific targeting of PFKFB3, as treatment of BDC2.5 splenocytes with the prototypical glycolysis inhibitor 2-Deoxy-D-glucose (2-DG), a non- metabolizable glucose analog, was only capable of dampening IFNγ secretion, but not IL-2 or TNFα (**Supplementary Figure 1**). However, activation of BDC2.5 splenocytes with two other known PFKFB3 inhibitors YN1 and PFK158 recapitulated our data with PFK15 (**Supplementary Figure 1**), further implicating glycolysis, and more specifically PFKFB3, as a vital metabolic pathway required for optimal activation and cytokine secretion by autoreactive effector T cells (**Figures 2F–H**).

### Targeting Glycolysis Delays the Onset of T1D in an Adoptive Transfer Model

**Figure 3:**
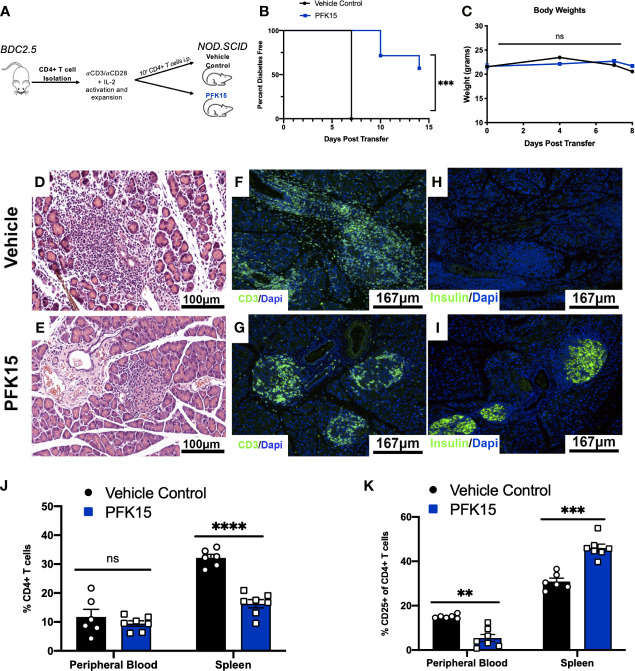
PFK15 treatment alters the diabetogenic potential of autoreactive CD4^+^ T cells and delays adoptive transfer of Type 1 Diabetes. The effect glycolysis inhibition has on Type 1 Diabetes onset was assessed using an adoptive transfer model. **(A)** Schematic diagram of experimental design for adoptive transfer studies. **(B)** Survival analysis of diabetes incidence in vehicle control and PFK15 treated groups. Kaplan-Meier survival analysis test was performed for statistical significance. **(C)** Body weight measurements. **(D, E)** Representative H&E staining to assess islet infiltration in pancreatic sections. **(F, G)** Representative pancreatic tissue immunostaining for the T cell marker CD3 co-stained with DAPI. **(H, I)** Representative pancreatic tissue immunostaining for insulin co-stained with DAPI. **(J)** Frequency of CD4^+^ T cells in the peripheral blood and spleens of PFK15 treated and control animals. **(K)** Frequency of CD4^+^ CD25^+^ T cells in the peripheral blood and spleens. All data are presented as the mean ± SEM. (n = 6-7 animals/group; (not significant (ns), **p < 0.01, ***p < 0.005, ****p < 0.0001)).

Based on the ability of PFK15 treatment to reduce BDC2.5 T cell responses, we examined the impact of glycolysis inhibition on the onset of diabetes using an adoptive transfer model (**Figure 3A**). Here, isolated CD4^+^ T cells from the spleens of NOD.BDC2.5.TCR.Tg animals were activated and expanded *ex vivo* with plate- bound αCD3/αCD28 and EL-4 supernatant as a source of IL-2 as previously described (28). We confirmed activation by measuring proinflammatory cytokine secretion in culture supernatants 3 days post activation, and observed significant levels of TNFα and IFNγ in BDC2.5 T cell cultures prior to adoptive transfer (**Supplementary Figure 2**). Activated T cells were transferred *via* intraperitoneal (i.p.) injection into immunodeficient NOD.*scid* recipients. A cohort of animals received 25 mg/kg of PFK15 treatment beginning on the day of the adoptive transfer. Animals were treated every other day for two weeks, and monitored for drug related toxicity (body weight) and diabetes onset (BG ≥ 350 mg/dl). 100% of control animals exhibited diabetes 7 days post-transfer, however PFK15 treatment delayed disease onset, with 57% of animals remaining diabetes free for the duration of the study, with no appreciable weight loss observed (**Figures 3B, C**). Pancreases from control animals exhibited invasive insulitis, while protected animals treated with PFK15 displayed peri- islet insulitis as observed *via* H&E staining (**Figures 3D, E**). In agreement with this, immunofluorescent (IF) staining for the T cell marker CD3 revealed reduced T cell infiltration into the islets of PFK15 treated animals corresponding to an inability for T cells to completely penetrate the islets (**Figures 3F, G**). As expected, loss of insulin staining was observed in diabetic controls, with retention of insulin staining observed in PFK15 treated animals, correlating with disease status (**Figures 3H, I**). Lastly, PFK15 treatment had no impact on circulating CD4^+^ T cell frequencies in the peripheral blood, however reduced CD4^+^ T cell percentages were observed in the spleens of treated animals, indicating reduced expansion of PFK15 treated T cells (**Figure 3J**). Analysis of CD25 on CD4^+^ T cells in control and PFK15 treated animals revealed a significant decrease in the percentage of CD4^+^ CD25^+^ T cells in the periphery, however a higher percentage of CD4^+^ T cells expressed CD25 in the spleens of treated animals compared to controls (**Figure 3K**). Although a larger percentage of CD4^+^ T cells in the spleens of treated animals expressed CD25, treated animals had less CD4^+^ T cell percentages in the spleens, indicating possible sequestration of effector- like T cells in the spleen compared to control animals, as glycolysis is required for proper T cell migration to sites of inflammation (29). Together, these data indicate that metabolic modulation by PFK15 treatment alters the diabetogenic potential of activated CD4^+^ T cells, thereby reducing the immunopathological parameters associated with disease onset.

### PFK15 Treatment Increases the Expression of Checkpoint Molecules PD-1 and LAG-3 on CD4^+^ T Cells

**Figure 4:**
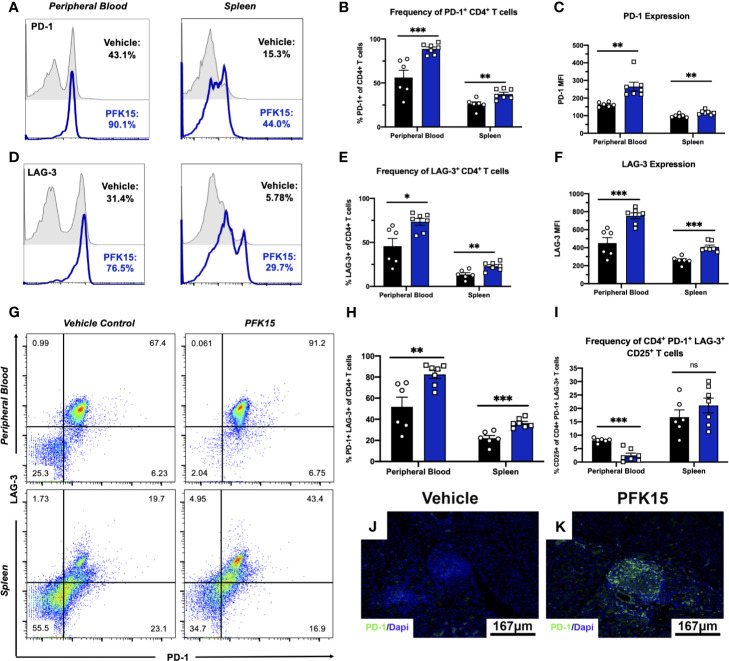
Inhibition of glycolysis results in increased expression of PD-1 and LAG-3 on CD4^+^ T cells. PD-1 and LAG-3 expression were assessed on CD4^+^ T cells in PFK15 treated and control animals. **(A, B)** Representative histogram and statistical analysis measuring the frequency of PD-1^+^ CD4^+^ T cells in the peripheral blood and spleen of PFK15 treated and control animals. **(C)** Statistical analysis of PD-1 expression (MFI) on CD4^+^ T cells in the peripheral blood and spleens. **(D, E).** Representative histogram and statistical analysis measuring the frequency of LAG-3^+^ CD4^+^ T cells in the peripheral blood and spleen of PFK15 treated and control animals. **(F)** Statistical analysis of LAG-3 expression (MFI) on CD4^+^ T cells in the peripheral blood and spleens of PFK15 and control animals. **(G, H)** Representative flow plots and statistical analysis measuring PD-1 and LAG-3 co-expression on CD4^+^ T cells in the peripheral blood and spleen of PFK15 treated and control animals. **(I)** Statistical analysis of the frequency of PD-1^+^ LAG-3^+^ CD4^+^ T cells expressing CD25. **(J, K)** Representative pancreatic tissue immunostaining for PD-1 co-stained with DAPI. All data are presented as the mean ± SEM. (n = 6-7 animals/group; (not significant (ns), *p < 0.05, **p < 0.01, ***p < 0.005)).

In the tumor microenvironment (TME), metabolic restriction leads to increased IR expression and subsequent T cell exhaustion (6). To investigate the mechanisms leading to T cell hyporesponsiveness and protection in PFK15 treated animals, we measured known markers of exhaustion, PD-1 and LAG-3, in the peripheral blood and spleens from control and PFK15 treated animals. Consistent with a hyporesponsive phenotype, PFK15 treatment led to significantly increased frequencies and expression of PD-1^+^ (**Figures 4A–C**) and LAG-3^+^ CD4^+^ T cells (**Figures 4D–F**) in the peripheral blood and spleens. Since T cell exhaustion is associated with increased expression of multiple immune inhibitory receptors (IRs), we assessed co-expression of PD-1 and LAG-3 and found that T cells from PFK15 treated animals retained high expression of both PD-1 and LAG-3 on circulating CD4^+^ T cells and T cells in the spleens (**Figures 4G, H**). Effector T cells transiently upregulate checkpoint molecules early during activation to temper their initial response and clonal burst (7, 30). To phenotypically characterize differences between PD-1^+^ LAG-3^+^ T cells in control and PFK15 treated animals, we measured the percentage of PD-1^+^ LAG-3^+^ T cells expressing the activation marker, CD25. Strikingly, we observed a higher percentage of PD-1^+^ LAG-3^+^ T cells in the peripheral blood of control animals expressing CD25, indicative of an effector phenotype, while PD-1^+^ LAG-3^+^ T cells in PFK15 treated animals had decreased CD25 expression, consistent with an exhaustion phenotype (**Figure 4I**). Similar to our analysis of CD25 on CD4^+^ T cells in **Figure 3K**, no differences in CD25 expression were observed in the spleen of control and PFK15 treated animals, providing further evidence for sequestration of effector like T cells due to the importance of both glycolysis and PD-1 signaling in T cell trafficking (29, 31). Finally, to determine the impact glycolysis inhibition had on T cell responses in the pancreas, we stained pancreatic sections for PD-1. Indeed, PFK15 treated animals displayed increased PD-1 staining in the pancreatic islets compared to diabetic control animals (**Figures 4I, J**). These results demonstrate that PFK15 treatment results in increased frequency of CD4^+^ T cells expressing checkpoint molecules PD-1 and LAG-3 *in vivo*, suggesting that inhibition of glycolysis induces potential T cell exhaustion thereby contributing to delayed T1D onset.

### Modulating Glycolysis Leads to Functional and Metabolic Exhaustion of Diabetogenic CD4^+^ T Cell Clones

**Figure 5:**
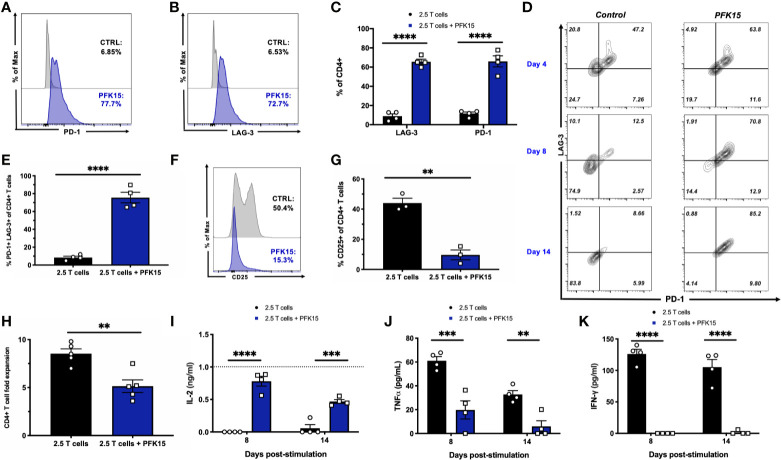
PFK15 treated T cell clones are functionally exhausted. BDC2.5 T cell clones were treated with PFK15 over the course of a 2- week restimulation period to perform mechanistic studies investigating whether glycolysis inhibition induces functional exhaustion of CD4^+^ T cells. **(A–C)** Representative histograms and statistical analysis assessing PD-1 and LAG-3 expression on control and PFK15 treated BDC2.5 T cell clones (n = 4). Cells were gated on CD4^+^ T cells. **(D)** Representative flow plots measuring PD-1 and LAG-3 co-expression on PFK15 treated and control BDC2.5 T cell clones on days 4, 8, and 14 post stimulation. Cells were gated on CD4^+^ T cells. **(E)** Statistical analysis of the percentage of BDC2.5 T cells co-expressing PD-1 and LAG-3 in control and treated flasks on Day 14 (n = 4). **(F, G)** Representative histogram and statistical analysis of the frequency of control and PFK15 treated BDC2.5 T cells expressing CD25 (n = 3). Cells were gated on CD4^+^ T cells. **(H).** Fold expansion of treated and control BDC2.5 T cells (n = 5). **(I-K).** ELISA analysis of IL-2, TNFα, and IFN*γ* in culture supernatants on days 8 and 14 post stimulation (n = 4). All data are presented as the mean ± SEM. (**p < 0.01, ***p < 0.005, ****p < 0.0001).

To further substantiate evidence of T cell exhaustion induction by PFK15 treatment, we performed mechanistic studies *in vitro* using the BDC2.5 T cell clone maintained on a 2-week restimulation schedule (22–25). This 2- week period allows for the treatment of T cells with PFK15 on a similar regimen to our *in vivo* study. Consistent with our *in vivo* data, sustained PFK15 treatment of BDC2.5 T cell clones significantly increased the expression of PD-1 and LAG-3 alone on CD4^+^ T cells (**Figures 5A–C**). However, while a hallmark of T cell exhaustion is the expression of checkpoint molecules, expression of these proteins alone is not indicative of exhaustion, as these molecules are upregulated transiently on the surface of newly activated T cells (7). We confirmed this by kinetically measuring PD-1 and LAG-3 co-expression on days 4, 8, and 14 post stimulation on CD4^+^ T cells from untreated and PFK15 treated flasks (**Figure 5D**), and quantified the percentage of CD4^+^ T cells co-expressing both PD-1 and LAG-3 on day 14 (**Figure 5E**). BDC2.5 T cells upregulated both PD-1 and LAG-3 early (Day 4) after activation, however by Day 8 most untreated CD4^+^ T cells began to downregulate expression of the checkpoint molecules as predicted, with an even further downregulation evident by Day 14 (**Figures 5D, E**) (30). Interestingly, PFK15 treated T cells displayed increased and sustained expression of PD-1 and LAG-3 over the course of the 14-day restimulation, with a majority of T cells expressing both PD-1 and LAG-3 on Day 14 (**Figures 5D, E**). Concomitant with this, PFK15 treated T cell clones had decreased expression of CD25 compared to control flasks, confirming that PD-1 and LAG-3 expression on PFK15 treated T cells occurred independently of late T cell activation (**Figures 5F, G**).

During exhaustion, progressive loss of function occurs in a hierarchical manner, with high proliferative capacity and IL-2 production lost first, followed by a reduced ability to produce TNFα and IFNγ (7, 30, 32). We assessed these parameters in order to link PD-1 and LAG-3 expression with functional measures of T cell fitness. While control BDC2.5 T cells expanded 9-fold, treated T cells proliferated significantly less, with a mean 5-fold expansion (**Figure 5H**). Coinciding with this, we measured IL-2 production in culture supernatants on days 8 and 14 post stimulation by ELISA and found significantly more IL-2 accumulated in cultures treated with PFK15 (**Figure 5I**), indicating a reduced ability to consume IL-2 as a growth factor compared to control T cell cultures (**Figures 5F, G**). Reduced consumption of IL-2 was likely due to the significantly decreased expression of the high affinity IL-2 receptor CD25 observed in PFK15 treated T cell cultures (**Figures 5F, G**). Further, the levels of IL-2 in PFK15 treated BDC2.5 cultures were similar to the amount of IL-2 supplemented into restimulation cultures from EL-4 supernatant (gray dotted line on the graph). We also measured the effector cytokines TNFα and IFNγ in cell culture supernatants on days 8 and 14 post stimulation, both of which were significantly reduced upon treatment with PFK15 (**Figures 5J, K**). In sum, these data indicate that targeting glycolysis leads to severe exhaustion of diabetogenic CD4^+^ T cell clones, and suggest this as the mechanism by which PFK15 treatment delays T1D onset *in vivo* (**Figure 3**).

**Figure 6:**
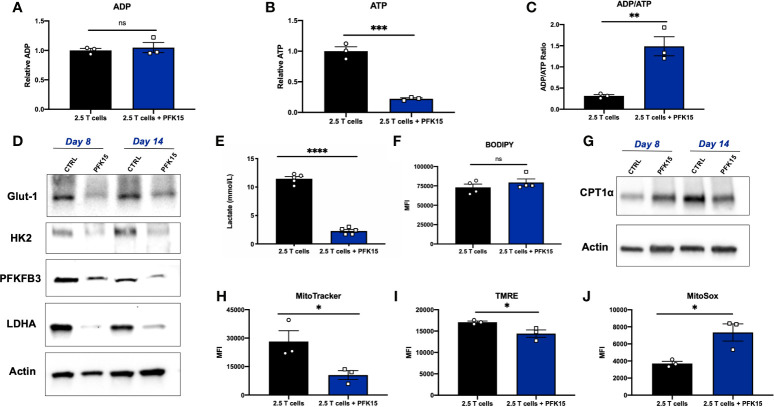
Glycolysis inhibition during activation renders CD4^+^ T cells metabolically insufficient. Assessing metabolic consequences of PFK15 treatment on BDC2.5 T cell clones. All analyses were performed 14 days post stimulation (Day 14), unless otherwise noted. **(A–C)** Relative ADP, ATP levels, and the ADP/ATP ratio were measured from Day 14 control and treated BDC2.5 T cells (n = 3). **(D)** Representative western blots for glycolysis proteins in control and PFK15 treated BDC2.5 T cell clones on days 8 and 14 post stimulation. **(E)** Lactate measurements in cell culture supernatants (n = 5). **(F)** Statistical significance of BODIPY fatty acid uptake of Day 14 control and PFK15 treated BDC2.5 T cells (n = 4). Cells were gated on CD4^+^ T cells. **(G)** Representative western blot analysis of CPT1α expression in control and PFK15 treated BDC2.5 T cell clones on days 8 and 14 post stimulation. Cells were gated on CD4^+^ T cells. **(H)** Statistical analysis of mitochondrial mass by MitoTracker green staining on Day 14 control and PFK15 treated T cell clones (n = 3). Cells were gated on CD4^+^ T cells. **(I)** Statistical analysis of mitochondrial membrane potential by TMRE staining on Day 14 control and PFK15 treated T cell clones (n = 3). Cells were gated on CD4^+^ T cells. **(J)** Statistical analysis of mitochondrial ROS by MitoSOX staining on Day 14 control and PFK15 treated T cell clones (n = 3). Cells were gated on CD4^+^ T cells. All data are presented as the mean ± SEM. (not significant (ns), *p < 0.05, **p < 0.01, ***p < 0.005, ****p < 0.0001).

Functional exhaustion of T cells is associated with downstream metabolic consequences. Generally speaking, exhausted T cells are thought to be metabolically deficient, with a majority of metabolic flux supporting cell survival, and limited reserve for fueling effector functions (33). As PFK15 treated T cell clones were functionally exhausted, we wanted to determine whether this phenotype correlated with decreased metabolic fitness as has been reported in the literature (33–35). To begin our investigation, we measured relative adenosine diphosphate (ADP) and ATP levels, and calculated the ADP/ATP ratio from Day 14 control and PFK15 treated T cell clones. While there was no difference in the relative levels of ADP, relative ATP levels were significantly reduced upon PFK15 treatment, indicating decreased metabolic flux (**Figures 6A, B**). While control T cells had a low ADP/ATP ratio, indicative of cell proliferation, PFK15 treated T cell clones had a significantly higher ADP/ATP ratio, indicating an inability to generate ATP efficiently and metabolic insufficiency (**Figure 6C**). To characterize the reduced metabolic capacity of PFK15 treated T cells, we measured indicators of glycolysis, fatty acid oxidation (FAO), and mitochondrial metabolism. As expected, PFK15 treated T cells had downregulated levels of the key glycolysis proteins Glut-1, HK2, PFKFB3, and LDHA and reduced supernatant lactate levels as compared to control BDC2.5 T cell clones, confirming an inability to engage in glycolysis upon encounter with β cell antigen (**Figures 6D, E**).

PD-1 signaling has been reported to promote FAO, with early exhausted T cells having increased carnitine palmitoyltransferase 1α (CPT1α) expression, a rate limiting enzyme that regulates mitochondrial fatty acid transport (36). To investigate FAO, we first measured fatty acid uptake utilizing a BODIPY C16 fluorescent analog, and found no significant difference in uptake of fatty acids in Day 14 control or PFK15 treated T cells (**Figure 6F**). CPT1α expression was measured by western blot analysis, and revealed increased expression of CPT1α in Day 8 PFK15 treated T cells, but decreased levels in treated Day 14 clones compared to untreated control T cells (**Figure 6G**). Together, these results indicate that by Day 8 control T cell clones are engaging in glycolysis in response to presentation of β cell antigen (**Figure 6D**) while PFK15 treated T cells are utilizing FAO. Although fatty acid uptake was unaltered, reduced CPT1α expression was observed in PFK15 treated clones compared to controls on Day 14, demonstrating a reduced ability to efficiently transport fatty acids into the mitochondria (**Figures 6F, G**), and likely contributing to the inefficient ATP generation we observed (**Figure 6B**).

A number of reports demonstrate that exhausted T cells have reduced mitochondrial fitness (34, 35, 37–40). To investigate the mitochondrial health of PFK15 treated T cells, we measured mitochondrial mass, reactive oxygen species (ROS), and mitochondrial membrane potential. Indeed, PFK15 treated T cell clones exhibited mitochondrial dysfunction as demonstrated by reduced mitochondrial mass (**Figure 6H**), decreased mitochondrial membrane potential (**Figure 6I**), and increased generation of mitochondrial ROS when compared to control T cells (**Figure 6J**). The mitochondrial dysfunction observed supports the reduced ATP levels measured in PFK15 treated T cells even when uptake of fatty acids was unaffected (**Figures 6F, G**), further pointing to inefficient utilization of nutrients and overall metabolic insufficiency. All in all, these data indicate that inhibition of glycolysis during the activation of autoreactive CD4^+^ T cell clones enforces an exhausted phenotype that mediates protection from T1D onset *in vivo*.

### PFK15 Treated CD4^+^ T Cells Are Terminally Exhausted

**Figure 7:**
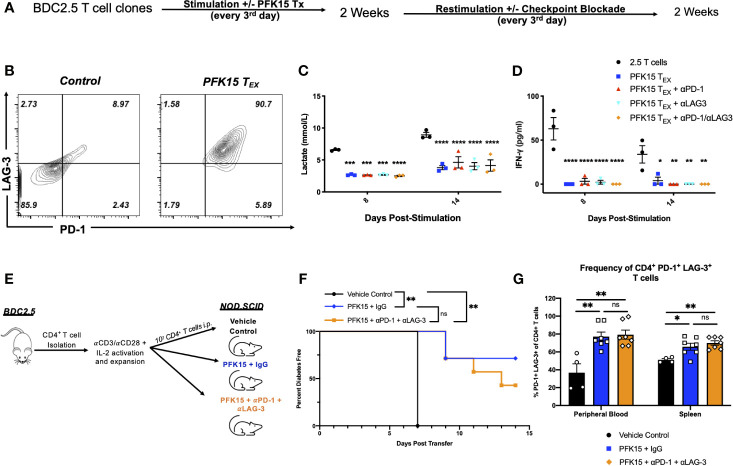
Inhibition of glycolysis leads to terminal exhaustion of CD4^+^ T cells that are refractory to checkpoint blockade. To determine the degree of exhaustion induced by PFK15 treatment, PFK15 treated T cell clones were restimulated ± checkpoint blockade. **(A)** Schematic diagram of experimental design for reinvigoration studies. **(B)** Representative flow plots of PD-1 and LAG-3 expression on CD4^+^ T cells on control and PFK15 T_EX_. **(C)** Statistical analysis of lactate secretion in Day 8 and 14 restimulation culture supernatants (n = 3). **(D)** ELISA analysis of IFN*γ* in culture supernatants on days 8 and 14 post stimulation (n = 3). **(E)** Schematic diagram of experimental design for *in vivo* reversibility studies. **(F)** Survival analysis of diabetes incidence in vehicle control and PFK15 treated groups. Kaplan-Meier survival analysis test was performed for statistical significance. **(G)** Statistical analysis measuring PD-1 and LAG-3 co-expression on CD4^+^ T cells in the peripheral blood and spleen of vehicle control (n = 4), PFK15 + IgG (n = 7), and PFK15 + αPD-1 + αLAG-3 (n = 7) treatment. All data are presented as the mean ± SEM. (not significant (ns), *p < 0.05, **p < 0.01, ***p < 0.005, ****p < 0.0001).

Exhausted T cell lineages display heterogeneity amongst subsets with unique characteristics and varying abilities to become reinvigorated (41, 42). To determine the state of exhaustion observed in PFK15 treated T cells, we performed reinvigoration experiments where a subset of BDC2.5 T cell clones were treated with PFK15 every third day for two weeks to induce exhaustion. Then, T cells from control or PFK15 treated flasks (PFK15 treated T cells put into restimulation cultures termed PFK15 T_EX_) were restimulated for another two weeks without further PFK15 treatment (**Figure 7A**). We first measured PD-1 and LAG-3 expression on PFK15 T_EX_ cells after restimulation and found that PFK15 T_EX_ sustained high expression of both PD-1 and LAG-3 compared to control cultures (**Figure 7B**), consistent with retention of an exhausted phenotype. Notably, PFK15 T_EX_ were unresponsive to IL-2 present in restimulation cultures, further confirming exhaustion and ruling out anergy (33). We also measured lactate and IFNγ in cell culture supernatants on days 8 and 14 post restimulation as indicators of re-engagement in the glycolysis pathway upon activation and effector function. We observed reduced lactate production and little secretion of IFNγ by PFK15 T_EX_ in response to restimulation, suggesting that treated T cell clones are terminally exhausted (**Figures 7C, D**).

Reports have demonstrated that terminally exhausted T cells are refractory to checkpoint blockade (33, 34, 37). To determine whether PFK15 T_EX_ cells were responsive to checkpoint blockade, a subset of restimulation cultures were treated with αPD-1, αLAG-3, or a combination of both αPD-1/αLAG-3 blocking antibodies as described in the methods. We measured lactate and IFNγ in cell culture supernatants to determine whether checkpoint blockade treatment would rescue PFK15 T_EX_ cell’s ability to utilize glycolysis and exert their effector function (**Figures 7C, D**). Neither αPD-1, αLAG-3, nor a combination of αPD-1 and αLAG-3 blocking antibody treatments were capable of rescuing PFK15 T_EX_ ability to respond to self- antigen, evidenced by significantly less lactate and IFNγ secretion measured in culture supernatants of PFK15 T_EX_ ± PD-1 and/or LAG-3 blockade compared to control T cells (**Figures 7C, D**). To confirm the induction of terminal exhaustion *in vivo*, we performed reversibility experiments using our adoptive transfer model, described in **Figure 3A**. Briefly, ex vivo activated BDC2.5 T cells were adoptively transferred into NOD.*scid* recipients, with recipient animals being separated into one of three treatment cohorts: 1) vehicle control, 2) PFK15 + IgG, and 3) PFK15 + αPD-1 + αLAG-3 (**Figure 7E**). Animals in the PFK15 treatment groups were treated every other day for two weeks, with IgG or checkpoint blockade treatment initiated in the second week. Notably, the dose of αPD-1 and αLAG-3 blocking antibodies administered have been previously shown to accelerate diabetes onset in NOD animals, therefore any delay or protection achieved with PFK15 treatment would be considered durable if irreversible with checkpoint blockade (20). As expected, 100% of control animals displayed fulminant diabetes by day 7 post- transfer, with significant delays associated with PFK15 treatment (**Figure 7F**). While 70% of animals receiving PFK15 + IgG remained diabetes free through the end of the study period, 42% receiving PFK15 + αPD-1 + αLAG-3 treatment were protected from disease (**Figure 7F**). Although more animals receiving checkpoint blockade succumbed to diabetes than PFK15 + IgG treated animals, diabetes incidence between these two groups were not statistically significant (p = 0.348), indicating an inability to reverse PFK15 induced T cell exhaustion. Finally, regardless of αPD-1 and αLAG-3 blockade, CD4^+^ T cells in PFK15 treated animals retained high expression of PD-1 and LAG-3 in the periphery and spleens compared to control animals, consistent with an exhausted phenotype (**Figure 7G**). Together, these data support the findings that inhibition of glycolysis in diabetogenic CD4^+^ T cells leads to terminal exhaustion, characterized by functional and metabolic dysfunction that is irreversible by restimulation or checkpoint blockade therapy *in vitro* and *in vivo*. This work demonstrates that the rate- limiting glycolysis enzyme PFKFB3 is a novel target for controlling autoreactive T cell activation as a means to protect against the onset of T1D by enforcing exhaustion of pathogenic T cells.

## Discussion

In the present study, we evaluated the ability of the anti- glycolytic PFK15 to control the activation of diabetogenic CD4^+^ T cells in T1D. To our knowledge, this is the first study testing the ability of a PFKFB3 inhibitor to prevent the onset of autoimmune diabetes. Our findings confirmed PFK15 inhibited glycolysis upon activation of CD4^+^ T cells (**Figure 1**), dampened autoreactive CD4^+^ T cell responses *in vitro* (**Figure 2**), and delayed disease onset *in vivo* (**Figures 3**, **4**). The protective benefits associated with PFK15 treatment are not entirely surprising as glycolysis is required for CD4^+^ T cell activation and IFNγ secretion. In fact, targeting T cell metabolism has been successfully used to prevent and reverse disease in other autoimmune diseases, albeit without mediating durable tolerance (14–17, 43). However, our findings demonstrate an induction of terminal exhaustion by glycolysis inhibition, which to our knowledge, has not been previously reported in the literature.

Previously, use of the prototypical glycolysis inhibitor 2-DG in SLE, RA, and MS failed to generate a long- lasting benefit, as cessation of treatment was associated with disease flare-ups (14–17). Dissimilarities in the observed outcomes between our study and others is likely due to differences in the mechanisms of inhibition. 2-DG is a glucose analog that indirectly targets the action of HK2 through competition with endogenous glucose levels (44). For this reason, an effective reduction in glycolytic flux requires high dose treatments, which are associated with adverse effects and non- specific targeting (44). In comparison, PFK15 is highly selective for a defined intracellular enzyme, thus requiring a much lower concentration for effective inhibition (44). These key differences appear to have drastically different outcomes on the T cell response, and the data included in **Supplementary Figure 1** confirm this, as treatment of BDC2.5 splenocytes with 2-DG *in vitro* was only able to reduce IFNγ secretion, and failed to recapitulate PFK15’s ability to dampen IL-2 and TNFα (**Supplementary Figure 1**). Strikingly, our data demonstrate an ability for PFK15 treated T cells to become early activated (**Figure 2D**). 2-DG treatment, however, leads to reduced CD69 expression upon activation, indicating maintenance of a quiescent phenotype that is reversible when treatment is stopped (17). The early activation observed in our model supports the need for T cells to lineage commit to effector subsets in order to induce a terminal phenotype. Finally, PD-1 and LAG-3 expression are induced upon TCR signaling, therefore early activation is required to upregulate IRs that ultimately render PFK15 treated T cells exhausted (**Figures 4**, **5**) (7, 30).

Although often overlooked, availability of nutrients is vital to maintaining T cell fitness. As described herein, T cells and cancer cells have a shared reliance on aerobic glycolysis, which becomes problematic in the TME when tumor cells metabolically restrict T cells, thus eliciting poor anti- tumor immunity (6, 34). Analogously, our data supports the idea that reduced glycolytic flux promotes T cell exhaustion, as PFK15 treatment induced defective effector responses upon activation (**Figures 2**, **5**). Glycolysis restriction, however, is not the only metabolic pathway dysregulated by TILs. Notably, TILs demonstrate a progressive loss of mitochondrial mass and function (34, 35, 37, 40). This, along with a low glucose environment, promote a state of metabolic insufficiency due to an inability to meet nutrient requirements; thus, leading to a permanent hyporesponsive state (6, 34, 35, 37, 40). Unexpectedly, we too observed mitochondrial dysfunction when glycolysis was inhibited (**Figure 6**). Although other factors in the TME contribute to repressed mitochondrial function, particularly hypoxia, our data strengthens the link between nutrient restriction and T cell exhaustion since glycolysis inhibition led to the development of metabolic insufficiency (45).

While the development of T cell exhaustion is detrimental in cancer and chronic infection, the opposite is true in autoimmunity, where induction of a hyporesponsive phenotype protects the host from attack (8). The onset of autoimmunity in T1D occurs due in large part to defective central and peripheral tolerance mechanisms that fail to control pathogenic T cells. This defect is due to dysregulated IR expression in T1D (46–48). In healthy individuals, binding of IR proteins to their associated ligand and subsequent downstream signaling act as a metaphorical “brake” that impedes T cell activation and protects against autoimmunity (7, 8, 30). Evidence of the importance of IRs is underscored by the accelerated diabetes observed in the absence or blockade of PD-1 or LAG-3 in NOD mice (20, 48, 49). Clinically speaking, polymorphisms in the PD-1 gene have been identified and associated with disease susceptibility (47). Concomitantly, T1D patients fail to upregulate PD-1 on T cells compared to control subjects, correlating to aberrant T cell activation and effector function (46, 50). In our study, PFK15 treatment led to increased PD-1 and LAG-3 expression on CD4^+^ T cells that correlated with protection from diabetes onset (**Figure 3**). Importantly, expression of IRs alone is not sufficient to induce T cell exhaustion, since activated T cells transiently upregulate both PD-1 and LAG-3 upon early activation (7, 30). In fact, exhaustion can occur even in the absence of checkpoint molecules, further complicating the role these molecules play in enforcing and maintaining functional exhaustion (34). While IRs may play a lesser role in driving exhaustion in other disease settings, our data reveal a pivotal role for PD-1 and LAG-3 in maintaining tolerance against autoimmune responses. In conclusion, we have demonstrated a unique ability to correct defects in peripheral tolerance mechanisms in T1D by inducing PD-1 and LAG-3 expression on CD4^+^ T cells. Increased IR expression and glycolysis restriction led to the functional and metabolic exhaustion of diabetogenic T cells independent of PD-1 and LAG-3 signaling, as checkpoint blockade failed to reverse this phenotype (**Figure 7**).

Therapeutic strategies for T1D have focused on two specific areas: 1) β cell replacement *via* regeneration of endogenous β cell mass or 2) immunomodulation (51, 52). Although innovative efforts have been made to restore β cell mass, these strategies ultimately fail due to reemergence of the autoimmune response (52). Moreover, although immunomodulation has yielded positive results in preclinical studies, success in the clinic has remained limited. Unfortunately, present clinical studies have only administered immunotherapies to patients with diagnosed T1D. By diagnosis, T1D patients have endured longstanding autoimmunity, with significant β cell loss, thus highlighting a need to intervene prior to symptomatic disease (1). Interestingly, the presence of autoantibodies is known to be a strong predictor for disease onset (53, 54). In fact, the first in man prevention study was published recently, where the αCD3 antibody teplizumab was utilized in patients at risk for diabetes development (51). Teplizumab delayed T1D onset by 2 years, and similarly to our study, protection was associated with increased expression of IRs and induction of a T cell hyporesponsive state (51). However broad immunosuppression was observed in teplizumab treated patients, which is an adverse effect of most immunotherapies due to non- specific targeting. A potential benefit of modulating glycolysis is the ability to specifically target activated T cells, while leaving established memory T cells and regulatory T cell populations unaffected based on their reliance on alternative metabolic pathways (4, 55–57). Clinically speaking, we would anticipate glycolysis inhibition to delay or prevent disease onset, while use of PFK15 in conjunction with methods to restore β cell mass may be a novel way to reverse T1D.

In summary, these findings demonstrate an ability to induce terminal exhaustion of autoreactive T cells in T1D by modulating the glycolysis pathway *via* targeting of PFKFB3. On a broader note, these data support a key role for glucose utilization in T cell activation and function, since an inability to efficiently metabolize glucose enforces a hyporesponsive phenotype. In our study, this phenotype was associated with expression of PD-1 and LAG-3, which are known to be dysregulated in T1D patients. This study remains focused on the ability to restrain diabetogenic CD4^+^ T cells due to their importance in the initiation of autoimmunity. While we would anticipate an overall benefit to other mediators of autoimmunity in T1D, like CD8^+^ T cells, further investigations are required to fully understand the impact glycolysis inhibition would have on other immune cell subsets. However, with the ability to restore tolerance in preclinical studies, we anticipate the use of metabolic modulators, like PFK15, may have a beneficial impact in both the clinical prevention and reversal of disease.

## Data Availability Statement

The raw data supporting the conclusions of this article will be made available by the authors, without undue reservation.

## Author Contributions

CM and JP conceived the project. CM, DP, KC, and JP, designed experiments. CM, LN, EO’C, DP, KC, and IT, performed experiments and/or analyzed data. KC and SS-L provided assistance, expertise, and reagents for histology experiments. CM wrote the manuscript; CM, DP, KC, SS-L, and JP contributed to and edited the manuscript. All authors contributed to the article and approved the submitted version.

## Funding

This work was supported by the Juvenile Diabetes Research Foundation (grant #: 2-SRA2020-910-S-B to JP), UPMC Children’s Hospital of Pittsburgh Foundation Cochrane Weber Endowed Fund (awarded to JP), and a UPMC Children’s Hospital of Pittsburgh Research Advisory Committee (RAC) Predoctoral Fellowship (awarded to CM).

## Conflict of Interest

The authors declare that the research was conducted in the absence of any commercial or financial relationships that could be construed as a potential conflict of interest.
