# Supplementary material for: Glycolysis Inhibition Induces Functional and Metabolic Exhaustion of CD4+ T Cells in Type 1 Diabetes
Source: Front Immunol. 2021 Jun 7;12:669456. doi: 10.3389/fimmu.2021.669456 (PMC8216385; doi:10.3389/fimmu.2021.669456)
Supplement: Supplementary file 1 [file DataSheet_1.docx]

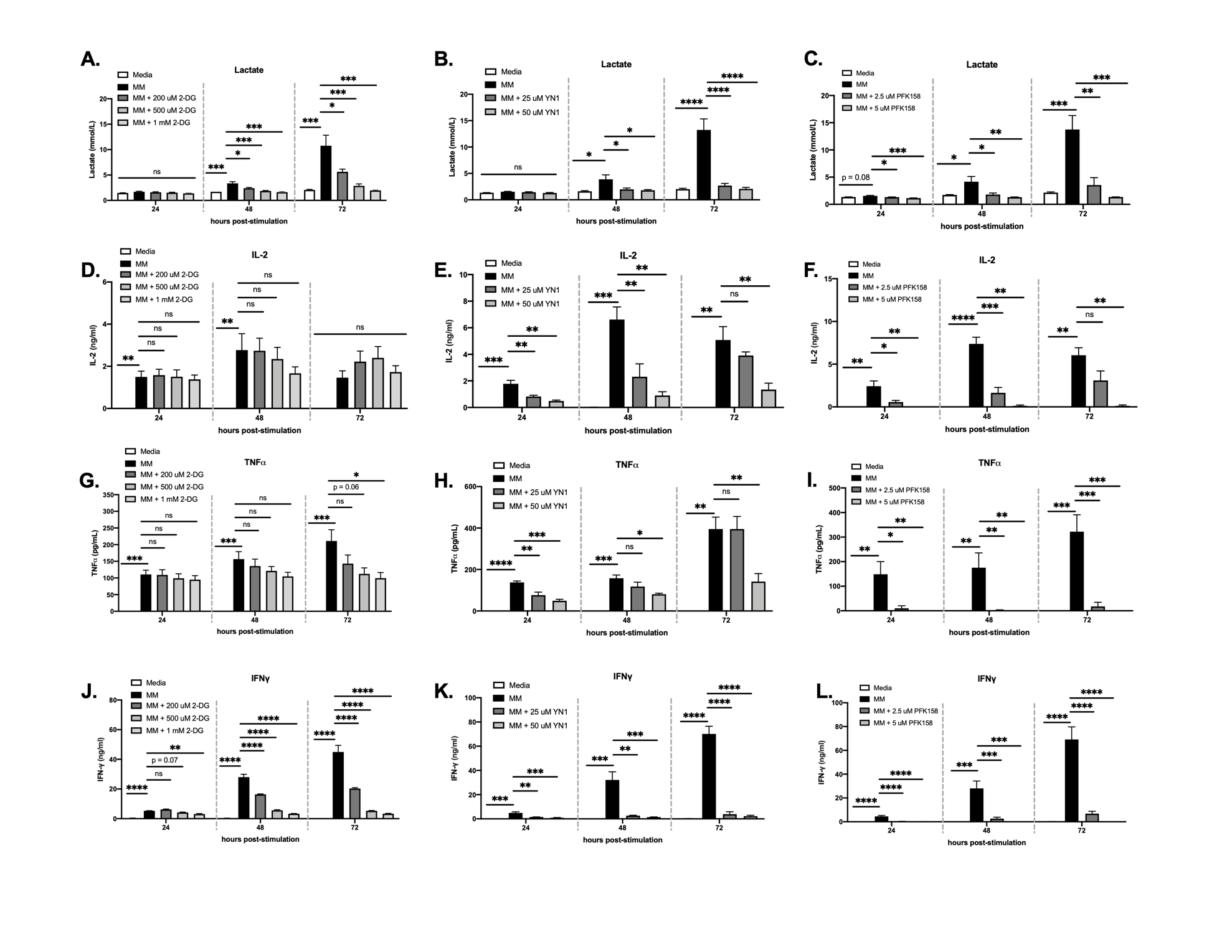
Supplementary Material

**Supplementary Figure 1.** **Specific targeting of PFKFB3, not glycolysis inhibition alone, reduces BDC2.5 T cell effector functions *in vitro*.** Comparing the impact of glycolysis inhibition with 2-DG or PFKFB3 inhibition (via administration of YN1 or PFK158) on diabetogenic CD4^+^ T cell responses *in vitro*. NOD.BDC2.5 splenocytes were stimulated with their cognate peptide MM ± 200μM, 500 μM, and 1mM 2-DG; 25-50 μM YN1, or 2.5-5 μM PFK158 for 24-72 hrs. **A-C.** Lactate measurements in 24- 72 hr cell culture supernatants from 2-DG (**A**), YN1 (**B**), and PFK158 (**C**) treated cultures. **D-F.** ELISA analysis of IL-2 in 2-DG (**D**), YN1 (**E**), and PFK158 (**F**) treated T cell culture supernatants 24-72 hrs post stimulation. **G-I.** ELISA analysis of TNFα in 2-DG (**G**), YN1 (**H**), and PFK158 (**I**) treated T cell culture supernatants 24-72 hrs post stimulation. **J-L.** ELISA analysis of IFNγ in 2-DG (**J**), YN1 (**K**), and PFK158 (**L**) treated T cell culture supernatants 24-72 hrs post stimulation. All data are presented as the mean ± SEM. (n = 3-5; * = p < 0.05, ** = p < 0.01, *** = p < 0.005, **** = p < 0.0001).


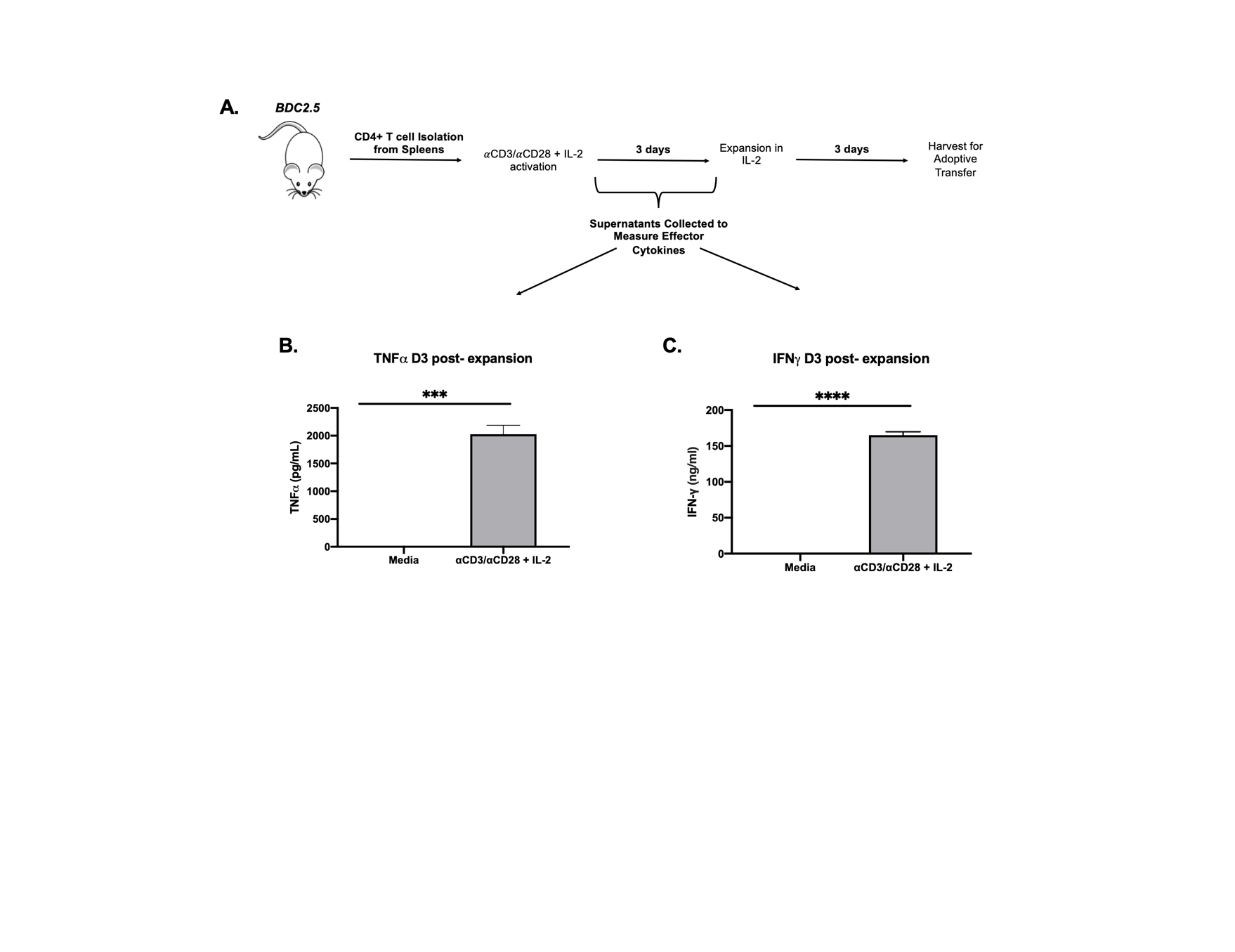


**Supplementary Figure 2.** ***ex vivo* activated BDC2.5 T cells produce proinflammatory cytokines TNFα and IFNγ** **prior to adoptive transfer.** Confirming the effector phenotype of *ex vivo* activated BDC2.5 T cells prior to adoptive transfer studies. Isolated CD4^+^ T cells from the spleens of NOD.BDC2.5.TCR.Tg animals were activated and expanded *ex vivo* with plate- bound ɑCD3/ɑCD28 and EL-4 supernatant as a source of IL-2. **A.** Schematic diagram of experimental design and timepoints for analysis. Cells in media alone served as controls. **B.** ELISA analysis of TNFα in BDC2.5 T cell culture supernatants 3 days post stimulation. **C.** ELISA analysis of IFNγ in BDC2.5 T cell culture supernatants 3 days post stimulation. All data are presented as the mean ± SEM. (n = 3; *** = p < 0.005, **** = p < 0.0001).
